# Re-evaluation of purse string suture in laparoscopic appendectomy

**DOI:** 10.1007/s00464-019-06828-5

**Published:** 2019-05-13

**Authors:** Kamleshsingh Shadhu, Dadhija Ramlagun, Yao Wang, Xiaochun Ping, Tao Chen, Yanhui Zhu, Zekuan Xu

**Affiliations:** 1 Department of General Surgery, The First Affiliated Hospital of Nanjing Medical University & Jiangsu Province Hospital, Guangzhou Road, 300, Gulou District, Nanjing, 210029 Jiangsu People’s Republic of China; 2 Division of Gastric Surgery, The First Affiliated Hospital of Nanjing Medical University & Jiangsu Province Hospital, Guangzhou Road, 300, Gulou District, Nanjing, 210029 Jiangsu People’s Republic of China; 3 Division of Breast Surgery, The First Affiliated Hospital of Nanjing Medical University & Jiangsu Province Hospital, Guangzhou Road, 300, Gulou District, Nanjing, 210029 Jiangsu People’s Republic of China; 4 Division of Colorectal Surgery, The First Affiliated Hospital of Nanjing Medical University & Jiangsu Province Hospital, Guangzhou Road, 300, Gulou District, Nanjing, 210029 Jiangsu People’s Republic of China

**Keywords:** Laparoscopic appendectomy, Intracorporeal knotting, Hem-o-lock polymeric clips, Purse string invaginating sutures, Acute appendicitis

## Abstract

**Purpose:**

The aim of this study was to compare the outcomes of laparoscopic appendectomy (LA) using purse string invaginating sutures (PS) with those using intracorporeal knotting (IK) or Hem-o-lock polymeric clips (HL).

**Methods:**

A total of 882 patients who underwent laparoscopic appendectomy from January 2015 to December 2017 were studied retrospectively. Of these, 538 patients used PS, 229 patients used IK and 115 patients used HL to close the appendiceal stump. Their demographic characteristics, intraoperative findings and postoperative complications were analysed retrospectively.

**Results:**

There were similar percentages of complicated cases in all the groups (21.7% in PS vs. 21.4% in IK vs. 24.3% in HL, *p* = 0.803). The mean length of hospital stay was shorter in PS group when compared to IK or HL group (3.72 + 2.35 in PS vs. 4.41 + 2.40 in IK, 4.43 + 2.66 in HL, *p* < 0.05) as well as lower ASA scores (1.7 + 0.6 in PS vs. 1.8 + 0.6 in IK vs. 1.7 + 0.6 in HL, *p* < 0.05). The overall complication rates for the PS, the HL and the IK groups were 12.1, 8.7 and 9.2%, respectively. The rate of wound infection was higher in PS group for uncomplicated appendicitis (5.0% in PS vs. 2.8% in IK and 1.1% in HL, *p* = 0.129). Furthermore, there were no differences in the rate of intra-abdominal infection among the groups in both uncomplicated and complicated cases.

**Conclusions:**

Based on our results, purse string suture failed to demonstrate better postoperative outcome in laparoscopic appendectomy and is no longer recommended by our institution as initial approach.

Recently, laparoscopy is commonly used in abdominal emergencies like acute appendicitis [1]. It has been shown that laparoscopic appendectomy (LA) has a lot of advantages as compared with open approach technique, including faster recovery, shorter hospital stays and lower percentage of surgical site infections [2]. There are several techniques to close appendicular stump during LA. However, a consensus about the optimal technique of the appendicular stump closure still seems unclear. There are several ways to close the stump of appendix, such as endo-loop, Hem-o-lock polymeric clips, intracorporeal knotting and even purse string suture. Purse string suture was a traditional procedure performed in open appendectomy. There are studies which did not argue in favour of purse string suture [3–5]. Nevertheless, its role in LA has not been examined due to its demanding technique. The aim of this study was to compare the outcomes of LA using purse string invaginating sutures (PS) with those using intracorporeal knotting (IK) or Hem-o-lock polymeric clips (HL).

## Materials and methods

Medical records of patients who underwent laparoscopic appendectomy from 1st January 2015 to 30th December 2017 at Jiangsu Province Hospital, the First Affiliated Hospital of Nanjing Medical University were retrospectively analysed. Patients who were younger than 14 years old, had concomitant surgery other than appendectomy, or had converted to open surgery, were excluded. 882 patients were identified and included in this study. All the subjects had been clinically diagnosed, met the indications for surgical treatment and signed the informed consent forms. All operations were performed by attending surgeons or senior residents who were experienced in laparoscopic appendectomy. The choice of surgical procedure for stump closure was determined by the surgeon’s preference.

The demographic characteristics of patients including age, gender, body mass index (BMI), initial body temperature, WBC count, time from the onset of right iliac fossa pain, ultrasound or CT scan positive for acute appendicitis, co-morbidities (diabetes, cardio-vascular diseases, cirrhosis, chronic renal disease, or immunosuppressive status), previous abdominal surgery were collected and analysed retrospectively. The intraoperative factors which include ASA score, operative time, intraoperative grading of appendicitis based on Gomes Score [6], number of complicated appendicitis including gangrenous, perforated, purulent appendicitis with abscess and/or localized/diffuse peritonitis and delayed appendicitis, intra-abdominal lavage and drain usage were reviewed.

After discharge, the patients were followed-up at the outpatient department at 1-week interval for estimation of complications and full recovery. Their postoperative outpatient records were reviewed, and telephone interviews were further carried out to ensure at least a follow-up period length of 6 months. Of the 882 patients, 732 responded to the telephone interview, 95 patients were tracked in their postoperative outpatient records, 55 patients (6.23%) were lost to follow-up. Any related complications that occurred during this postoperative period were included in the analysis. This study was approved by hospital institutional review board.

### Surgical technique

Prior to surgery, all the patients received one dose of second-generation cephalosporin for antibiotic prophylaxis. Laparoscopic appendectomy was performed using classic three port technique. Prior to inserting the trocar, a Foley catheter was inserted into the bladder. A 10-mm trocar (Johnson and Johnson’s, USA) was then inserted under the umbilicus using the open technique. Pneumoperitoneum was applied with carbon dioxide (CO_2_), and intra-abdominal pressure was fixed at 10–12 mmHg. Next, a 5-mm trocar was inserted into the suprapubic area before applying a 10-mm trocar to the right iliac fossa under direct vision. A rigid 30 degree 10-mm laparoscope and standard rigid 5-mm laparoscopic instruments were used. Patients were placed in reverse Trendelenburg position with left tilt. Distal ileum was pushed to the left side of the abdomen to help expose the appendix. After the appendix became visible, it was lifted from the mesoappendix. A hole was made in the mesoappendix and an absorbable clip was applied through before dividing the artery. The base of appendix was managed as described below.

### Description of intracorporeal technique

**Fig. 1:**
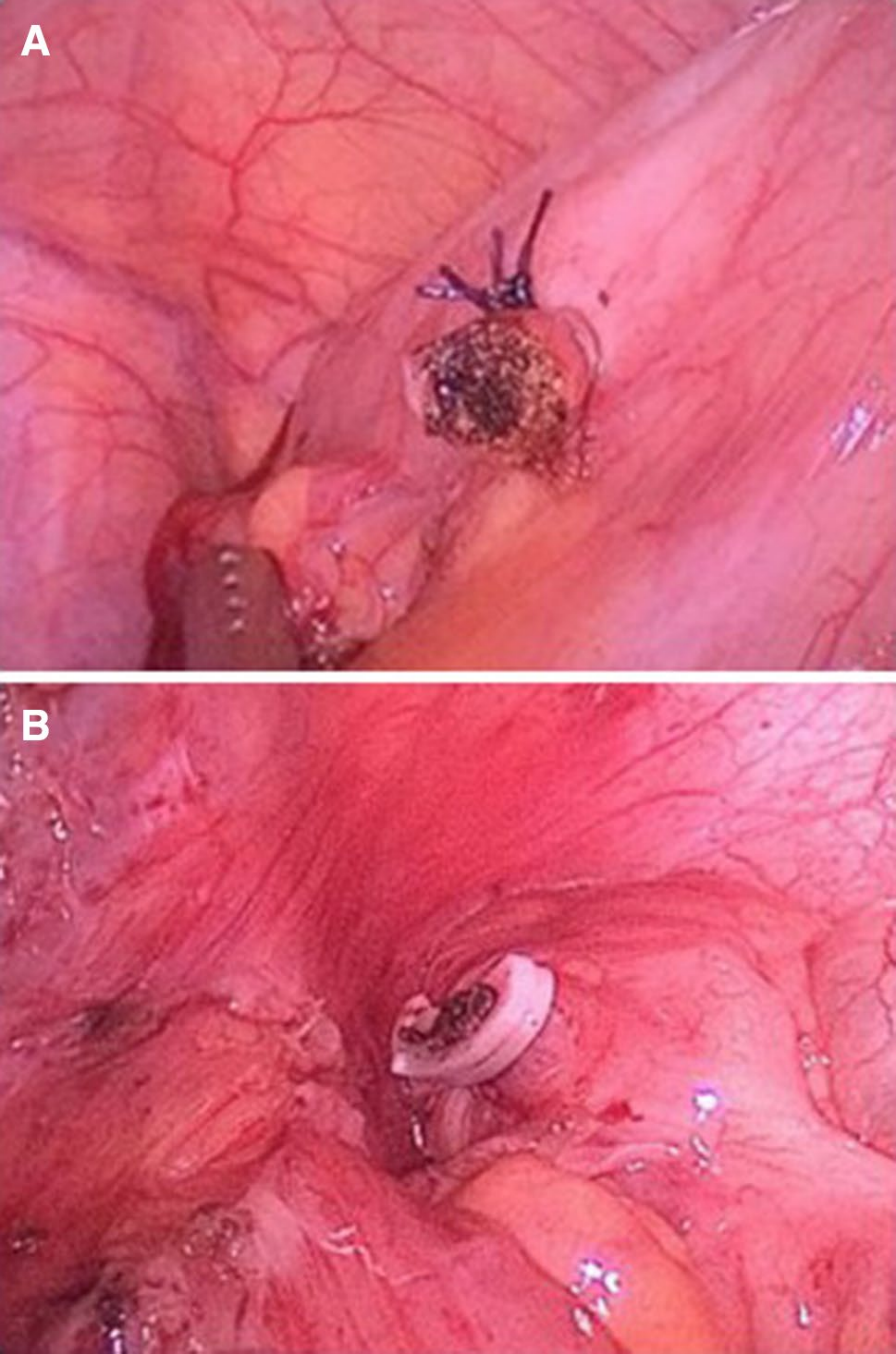
**A** The stump of the appendix base by double knotting after removal of specimen through a 10 mm trocar via laparoscopic endo-bag. **B** Application of Hem-o-lock polymeric clips at the base of the appendix

In the case of intracorporeal knotting, a 3/0 vicryl string (Johnson and Johnson’s) was passed around the base of appendix, two square knots were made intracorporeally to ligate the appendix. The distal part of the appendix was resected 0.5 cm distal to the knots. The specimen was removed through the 10 mm trocar using a laparoscopic endo-bag (Fig. 1A).

### Description of Hem-o-lock polymeric clips

In the case of Hem-o-lock polymeric clips, two clips were applied at the base of the appendix instead of knots (Fig. 1B). In few cases when the width of the base was beyond the length of the jar of clip, an intracorporeal knot was first applied at the base to reduce the diameter of the appendiceal base.

### Description of purse-string suture

**Fig. 2:**
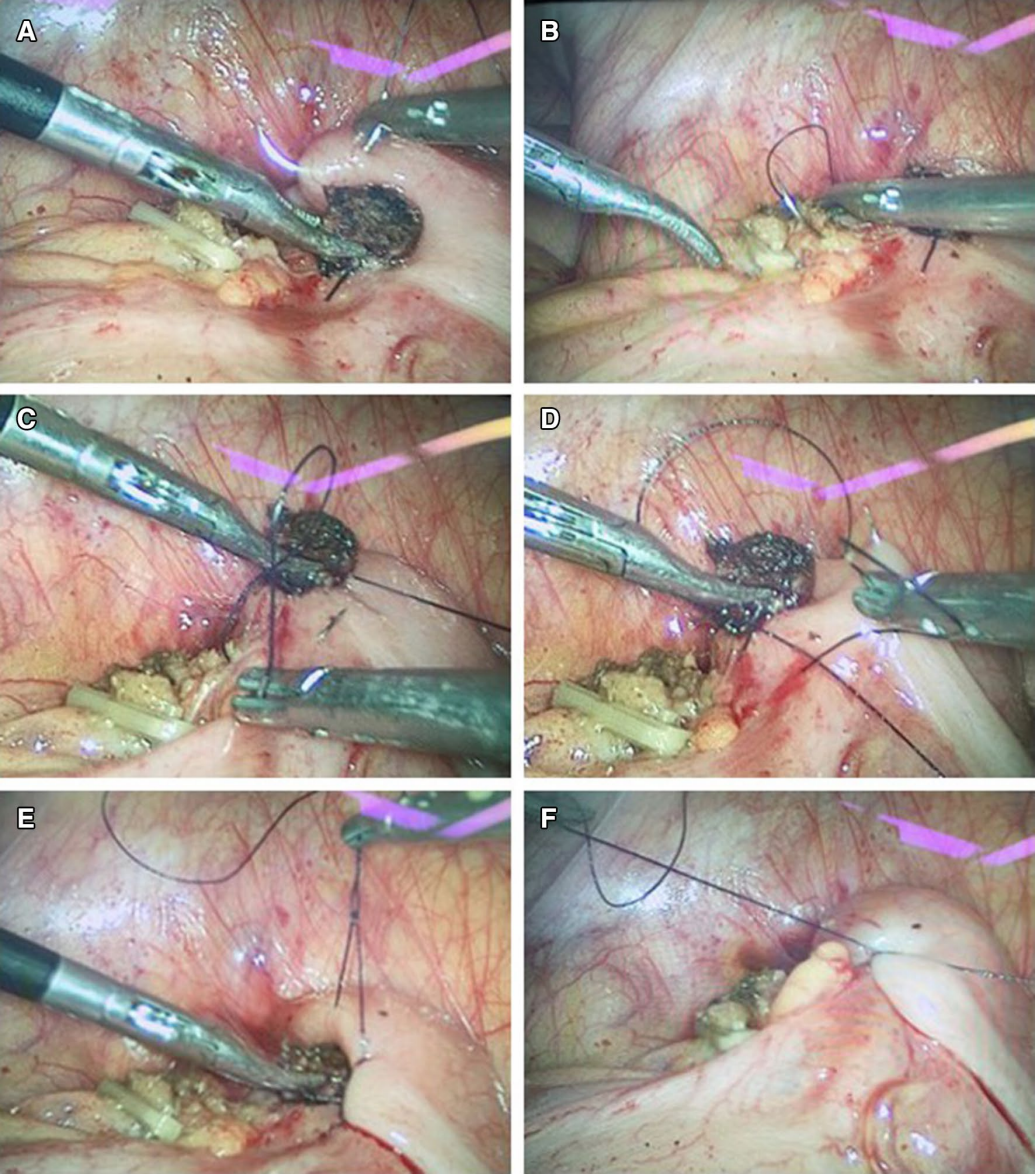
A counter-clockwise purse string suture was made 0.5–1.0 cm around the base of the appendix. The base was pushed into the purse while lifting the first knot. The second knotting was made to finalise the purse-string suture

In the case of purse string suture, two intracorporeal knots were made at the base of appendix and the appendix was resected as described above, leaving the stump of the appendix. Then, a purse-string suture by 3/0 vicryl was made 1.0 cm around the base of the appendix. When the first knot was formed but not secured, two ends of the string was lifted by the grasper through the 10 mm trocar, the other grasper through the 5 mm trocar then pushed the stump into the cecum. Once the stump was fully invaginated into the cecum, the first knot was secured. Then, the second knotting was made to finalise the purse-string suture (Fig. 2).

After removal of the specimen, abdominal lavage, using saline and drainage, was performed by the surgeon’s judgement. After the operation, patient received continuous antibiotics before discharge and could have liquid diet if tolerated. Patients were discharged once their body temperature and white blood cell count returned to normal and can tolerate normal diet.

### Statistical analyses

Statistical analysis was performed using the statistical software package SPSS 21.0. Calculated data was represented by “mean ± SD”. The anova and *t* test was used for comparison between the groups; the count data use case number (*n*) indicates and the comparison between the count data group rate (%) was performed using the *χ*^2^ test or Fisher’s exact test, and the two-sided test level was set to be *α* = 0.05. The difference was statistically significant at *p* < 0.05.

## Results

Eight hundred and eighty-two patients were included in this study. Among them, 458 were females and 424 were males. They were divided into three groups based on the techniques of stump closure. There were 538 patients in the purse string suture group (PS), 229 in the intracorporeal knotting group (IK) and 115 in the Hem-o-lock polymeric clips group (HL). Their demographic characteristics are summarized in Table 1.

**Table 1:** Demographic results of patients

	Intracorporeal knotting	Hem-o-lock	Purse string suture	*p* value
	*n* = 229	*n* = 115	*n* = 538	
Age (years)	44.4 + 18.3	42.8 + 20.6	41.1 + 18.7	0.083
Female gender (*n*)	124 (54.1%)	58 (50.4%)	276 (51.3%)	0.706
BMI	23.0 + 3.4	22.2 + 3.2	22.6 + 3.5	0.288
Initial body temperature (celsius)	37.3 + 0.7	37.3 + 0.9	37.2 + 0.7	0.494
White blood cell count	13.5 + 4.8	13.4 + 4.4	13.3 + 4.4	0.909
Time to onset of pain (days)	1.3 + 1.0	1.5 + 1.0	1.5 + 1.3	0.201
Co-morbidities (*n*)	53 (23.1%)	28 (24.3%)	116 (21.6%)	0.763
Previous abdominal surgery (*n*)	22 (9.7%)	10 (8.8%)	65 (12.3%)	0.406
Diagnostic imaging				0.007*
CT scan	122 (53.7%)	58 (50.4%)	239 (44.8%)	
Ultrasound	86 (37.9%)	48 (41.7%)	206 (38.6%)	

*p < 0.05

There were no significant differences in age, gender, BMI, initial body temperature, white blood cell count, co-morbidities, and previous abdominal surgery among three groups. Patients in the PS group had less CT scans prior to surgery (44.8% in PS vs. 53.7% in IK vs. 50.4% in HL, *p* < 0.05). They also had the lowest ASA score when compared to the other two groups (1.7 ± 0.6 in PS vs. 1.8 ± 0.6 in IK vs. 1.8 ± 0.6 in HL, *p* < 0.05) (Table 2).

**Table 2:** Characteristics of hospital stay

	Intracorporeal knotting	Hem-o-lock	Purse string suture	*p* value
	*n* = 229	*n* = 115	*n* = 538	
ASA score	1.8 + 0.6	1.8 + 0.6	1.7 + 0.6	0.040*
Complicated appendicitis^a^ (*n*)	49 (21.4%)	28 (24.3%)	117 (21.7%)	0.803
Laparoscopic grading system of acute appendicitis according to Gomes Score^b^				0.360
0	12 (5.2%)	4 (3.5%)	18 (3.3%)	
1	95 (41.5%)	48 (41.7%)	272 (50.6%)	
2	75 (32.8)	36 (31.3%)	143 (26.6%)	
3a	22 (9.6%)	19 (16.5%)	60 (11.2%)	
3b	12 (5.2%)	4 (3.5%)	20 (3.7%)	
4a	6 (2.6%)	1 (0.9%)	7 (1.3%)	
4b	2 (0.9%)	1 (0.9%)	9 (1.7%)	
5	5 (2.2%)	2 (1.7%)	9 (1.7%)	
Operative time (min)	76.0 + 46.5	74.3 + 33.8	71.4 + 44.6	0.400
Intraoperative lavage (*n*)	64 (27.9%)	23 (20.0%)	137 (25.5%)	0.279
Lavage volume (ml)	141.4 + 96.5	223.9 + 194.7	149.3 + 129.1	0.025*
Drain usage (*n*)	112 (48.9%)	44 (38.3%)	208 (38.7%)	0.024*
Length of drainage (days)	2.97 + 1.57	3.12 + 1.81	2.92 + 1.64	0.772
Hospital stay (days)	4.41 + 2.40	4.43 + 2.66	3.72 + 2.35	0.001*

*p < 0.05

^a^Complicated appendicitis: gangrenous, perforated, purulent appendicitis with abscess and/or localized/diffuse peritonitis and delayed appendicitis

^b^Gomes Score: the appendix was graded as to different levels based upon its visual appearance: grade 0 (normal looking), 1 (redness and edema), 2 (fibrin), 3a (segmental necrosis), 3b (base necrosis), 4a (abscess), 4b (regional peritonitis), and 5 (diffuse peritonitis)

In terms of severity of inflammation, the suppurative type was the most frequent type and there were similar percentages of complicated appendicitis in all the groups (21.7% in PS vs. 21.4% in IK vs. 24.3% in HL, *p* = 0.803). There were no significant differences in operative time and intraoperative lavage among the groups. Though those in the HL group who had the lavage had the highest volume (223.9 ml in HL vs. 149.3 ml in PS and 141.4 ml in IK, *p* < 0.05). Patients in the PS and HL groups had similar percentage of drainage usage, lower than those in the IK group (38.7% in PS vs. 38.3% in HL vs. 48.9% in IK, *p* < 0.05). All the groups kept the drainage for similar numbers of days, but the PS group had shortest length of hospital stay when compared to the other groups (3.73 ± 2.35 days in PS vs. 4.41 ± 2.40 days in IK vs. 4.43 ± 2.66 days in HL, *p* < 0.05).

The overall complication rates for the PS, the HL and the IK groups were 12.1, 8.7 and 9.2%, respectively (Table 3). There were no significant differences in the rate of wound and intra-abdominal infection and postoperative pain among the groups. One patient in the PS group had postoperative intra-abdominal bleeding and one patient in the HL group had postoperative pneumonia, both of which were managed by medical treatments. Two patients in the IK group and four patients in the PS group readmitted for intra-abdominal infection. Image-guided percutaneous drainages were used for postoperative intra-abdominal abscess (one patient in IK, two in HL, and one in PS, respectively). One patient in the PS group had reoperation 2 months postoperatively for adhesive small bowel obstruction. There was one mortality in the PS group, who was a 79-year-old female died of severe sepsis caused by postoperative intra-abdominal abscess.

**Table 3:** Overall complications

	Intracorporeal knotting	Hem-o-lock	Purse string suture	*p* value
	*n* = 229	*n* = 115	*n* = 538	
Complications	21 (9.2%)	10 (8.7%)	65 (12.1%)	0.387
Wound infection	10 (4.4%)	4 (3.5%)	29 (5.4%)	0.631
95% confidence interval	[1.7–7.0%]	[0.1–6.9%]	[3.5–7.3%]	
Intra-abdominal infection	6 (2.6%)	5 (4.3%)	17 (3.2%)	0.676
95% confidence interval	[0.5–4.7%]	[0.6–8.1%]	[1.7–4.6%]	
Bowel obstruction	0 (0.0%)	0 (0.0%)	1 (0.2%)	1.000
Prolonged abdominal pain	5 (2.2%)	1 (0.9%)	14 (2.6%)	0.676
Prolonged diarrhoea	0 (0.0%)	0 (0.0%)	5 (0.9%)	0.326
Others	0 (0.0%)	1 (0.9%)	1 (0.2%)	0.309
Image-guided percutaneous drainage	1 (0.4%)	2 (1.7%)	1 (0.2%)	0.062
Readmission	2 (0.9%)	0 (0.0%)	4 (0.7%)	0.858
Reoperation	0 (0.0%)	0 (0.0%)	1 (0.2%)	1.000
Mortality	0 (0.0%)	0 (0.0%)	1 (0.2%)	1.000

The subgroup analysis of uncomplicated and complicated appendicitis was shown in Tables 4 and 5. Compared to the IK and the HL groups, patients in the PS group had the highest rate of wound infection for uncomplicated appendicitis (5.0% in PS vs. 2.8% in IK vs. 1.1% in HL, *p* = 0.129), but the difference was not significant. They also had the lowest ASA score (1.6 ± 0.6 in PS vs. 1.7 ± 0.6 in IK vs. 1.8 ± 0.6 in HL, *p* < 0.05), longest duration of pain before surgery (1.5 ± 1.4 in PS vs. 1.1 ± 0.8 in IK and 1.4 ± 1.0 in HL, *p* < 0.05), the least use of drainage (28.7% in PS vs. 40.6% in IK vs. 29.9% in HL, *p* < 0.05) and the shortest length of hospital stay (3.3 ± 2.1 in PS vs. 4.1 ± 2.3 in IK vs. 3.6 ± 1.7 in HL, *p* < 0.05). As for complicated appendicitis, the rates of wound infection and abdominal infection were similar among the groups.

**Table 4:** Uncomplicated appendicitis

	Intracorporeal knotting	Hem-o-lock	Purse string suture	*p* value
	*n* = 180	*n* = 87	*n* = 421	
Age (years)	42.3 + 17.2	41.6 + 19.0	40.2 + 18.6	0.425
Female gender (*n*)	95 (52.8%)	46 (52.9%)	219 (52.0%)	0.980
BMI	23.0 + 3.5	22.0 + 3.2	22.4 + 3.4	0.169
Initial body temperature (celsius)	37.2 + 0.7	37.2 + 0.8	37.1 + 0.6	0.383
White blood cell count	13.5 + 4.9	13.4 + 4.6	13.2 + 4.3	0.794
Time to onset of pain (days)	1.1 + 0.8	1.4 + 1.0	1.5 + 1.4	0.005*
Co-morbidities (*n*)	42 (23.3%)	19 (21.8%)	83 (19.7%)	0.592
Previous abdominal surgery (*n*)	17 (9.4%)	8 (9.2%)	54 (13.1%)	0.336
ASA score	1.7 + 0.6	1.8 + 0.6	1.6 + 0.6	0.030*
Operative time (min)	75.5 + 49.3	71.0 + 32.5	68.1 + 45.4	0.193
Intraoperative lavage (*n*)	48 (26.7%)	16 (18.4%)	95 (22.6%)	0.295
Lavage volume (ml)	130.2 + 91.5	212.5 + 205.3	118.5 + 92.4	0.007*
Drain usage (*n*)	73 (40.6%)	26 (29.9%)	121 (28.7%)	0.016*
Length of drainage (days)	2.7 + 1.5	2.6 + 1.6	2.6 + 1.3	0.845
Hospital stay (days)	4.1 + 2.3	3.6 + 1.7	3.3 + 2.1	0.001*
Complications	14 (7.8%)	4 (4.6%)	46 (10.9%)	0.129
Wound infection	5 (2.8%)	1 (1.1%)	21 (5.0%)	0.191
95% confidence interval	[0.4–5.2%]	[0–3.4%]	[2.9–7.1%]	
Intra-abdominal infection	4 (2.2%)	2 (2.3%)	7 (1.7%)	0.776
95% confidence interval	[0.1–4.4%]	[0–5.5%]	[0.4–2.9%]	
Readmission	2 (1.1%)	0 (0.0%)	1 (0.2%)	0.858
Reoperation	0	0	0	
Mortality	0	0	0	

*p < 0.05

**Table 5:** Complicated appendicitis

	Intracorporeal knotting	Hem-o-lock	Purse string suture	p value
	*n* = 49	*n* = 28	*n* = 117	
Age (years)	52.3 + 20.1	46.5 + 22.8	44.5 + 18.4	0.064
Female gender (*n*)	29 (59.2%)	12 (42.9%)	57 (48.7%)	0.320
BMI	22.8 + 2.9	22.7 + 3.5	23.3 + 3.7	0.741
Initial body temperature (celsius)	37.6 + 0.8	37.6 + 1.0	37.6 + 0.8	0.990
White blood cell count	13.6 + 4.4	13.4 + 3.7	13.9 + 4.6	0.885
Time to onset of pain (days)	2.1 + 1.4	1.8 + 1.0	1.6 + 1.0	0.057
Co-morbidities (*n*)	11 (22.4%)	9 (32.1%)	33(28.2%)	0.619
Previous abdominal surgery (*n*)	5 (10.6%)	2 (7.4%)	11 (9.4%)	0.939
ASA score	2.0 + 0.6	1.8 + 0.7	1.9 + 0.6	0.491
Operative time (min)	78.1 + 34.3	85.3 + 36.5	83.4 + 38.6	0.653
Intraoperative lavage (*n*)	16 (32.7%)	7 (25.0%)	42 (35.9%)	0.542
Lavage volume (ml)	175.0 + 106.4	250.1 + 180.0	219.0 + 168.0	0.506
Drain usage (*n*)	39 (79.6%)*	18 (64.3%)	87 (74.4%)	0.335
Length of drainage (days)	3.3 + 1.4	4.0 + 1.4	3.2 + 1.9	0.371
Hospital stay (days)	5.5 + 2.3	6.9 + 3.3	5.1 + 2.5	0.006*
Complications	7 (14.3%)	6 (21.4%)	19 (16.2%)	0.702
Wound infection	5 (10.2%)	3 (10.7%)	8 (6.8%)	0.612
95% confidence interval	[1.7–18.6%]	[0–22.1%]	[2.2–11.4%]	
Intra-abdominal infection	2 (4.1%)	3 (10.7%)	10 (8.5%)	0.506
95% confidence interval	[0–9.6%]	[0–22.1%]	[3.4–13.6%]	
Readmission	0 (0.0%)	0 (0.0%)	3 (2.6%)	0.720
Reoperation	0	0	1 (0.9%)	1.000
Mortality	0	0	1 (0.9%)	1.000

*p < 0.05

## Discussion

The most frequently used surgical techniques to close the stump during LA are an intracorporeal knotting (IK), Hem-o-lock polymeric clips (HL) and purse string suture (PS) [7, 8]. However, it has been stated by prospective studies and meta-analysis that these techniques do not show any significant superiorities to each other in terms of surgery time, pre-operative and postoperative complication rates and length of hospital stay [9, 10]. In our study, we failed to show that PS was superior to IK or HL techniques in terms of complication rate and infection rate. We found purse string suture increased the rate of wound infection, especially among uncomplicated appendicitis. The confidence interval overlaps the null hypothesis so the *p* value would be insignificant, but the actual magnitude of the interval demonstrates that in fact most of the interval lies above the null, showing how PS technique is not recommended for this specific reason.

Despite of the retrospective nature of this study, most factors among the groups for uncomplicated appendicitis were comparable. The only few differences were that patients in the PS group had the lowest ASA score and shortest hospital stay. The topic of the use of shortened postoperative antibiotic may cause more incisional infection, is still under debate [11]. Yet, single-dose prophylaxis or prophylaxis ending within 24 h after operation is recommended by guidelines. Prolonged postoperative dosing of antibiotics does not provide additional benefits and is associated with increased risk of adverse events and induction of antimicrobial resistance [12]. The application of purse string suture with invagination into cecum to close appendicular stump during LA is much more demanding and requires some experience in laparoscopic sewing [13]. In some severe cases, purse string suture was even not applicable due to oedema of the cecum. Yet, our study failed to show that purse string suture provides any improvement in terms of intra-abdominal infection.

In the IK group, we observed a higher rate of drainage and longer hospital stay. This may be attributed to the low confidence of surgeons about security of the knotting. Indeed, it has been suggested to surgeons in a study by Gonenc et al. [14], to perform the IK technique to close the appendix stump in LA with an experienced surgeon on first cases [9, 10, 14, 15]. Moreover, studies have shown that the usage of drains have prolonged hospital stays [16, 17].

The Hem-o-lock polymeric clips technique was found to be feasible, safe, and cost-effective ligation technique of the appendicular stump [18–21]. Nevertheless, the safe use of Hem-o-lock polymeric clips is significantly limited by the maximum diameter of the closing appendix of 10 mm. Usually the inflamed appendix is thicker which may create difficulty in their application [4]. As it is the case in our study, its usage was limited in 13.0% of patients in our cohort. However, the Hem-o-lock clips technique provided the similar results compared to the intracorporeal knotting and purse string suture.

In addition, studies have shown that there has been no intraoperative complications and no mortality among the three groups of patients [13, 14, 22–25]. However, in our study we did have mortality in the PS group of patients which did not guarantee its’ safety.

This retrospective study has several limitations. First, our study was not prospectively designed. 6.23% out of the total number patients were lost to follow-up though they were evenly distributed among the groups (data not shown). The groups were not homogeneously divided to be compared with each other. As a teaching hospital, most of the studied cases were performed by senior residents and junior surgeons. Despite the effort to include surgeons who had performed at least 25 LA, surgeons who were included in our study were heterogeneous in their experiences and judgements. This may explain higher infection rates in the complicated cases in this study. Thus, further study which only include few senior surgeons are needed. Second, as required by hospital and insurance policy, LA with endo-loop or staple was not performed in our hospital and therefore not included in this study. Lacking such a data may further prevent comparing results with studies including those methods. Nevertheless, our study showed similar results compared to those with IK and Hem-o-lock techniques.

## Conclusion

Our study did not find any benefit of purse string suture in laparoscopic appendectomy in terms of postoperative infection rates and our institution has begun to consider other techniques like Hem-o-lock over PS as initial approach. Therefore, we do not recommend purse string suture in laparoscopic appendectomy.

## References

[1] Original article open or laparoscopic appendectomy?

[2] Thomson JE (2015). Laparoscopic versus open surgery for complicated appendicitis: a randomized controlled trial to prove safety. Surg Endosc.

[3] Kazemier G (2006). Securing the appendiceal stump in laparoscopic appendectomy: evidence for routine stapling?. Surg Endosc Other Interv Tech.

[4] Partecke LI (2010). Laparoscopic appendectomy using a single polymeric clip to close the appendicular stump. Langenbecks Arch Surg.

[5] Delibegović S, Matović E (2009). Hem-o-lok plastic clips in securing of the base of the appendix during laparoscopic appendectomy. Surg Endosc.

[6] Augusto GC (2015). Acute appendicitis: proposal of a new comprehensive grading system based on clinical, imaging and laparoscopic findings. World J Emerg Surg.

[7] Sohn M (2014). Stump closure in laparoscopic appendectomy. Influence of endoloop or linear stapler on patient outcome. Chirurg.

[8] Delibegović S (2012). The use of a single Hem-o-lok clip in securing the base of the appendix during laparoscopic appendectomy. J Laparoendosc Adv Surg Tech A.

[9] Sajid MS (2009). Use of endo-GIA versus endo-loop for securing the appendicular stump in laparoscopic appendicectomy: a systematic review. Surg Laparosc Endosc Percutan Tech.

[10] Ates M (2012). Comparison of intracorporeal knot-tying suture (polyglactin) and titanium endoclips in laparoscopic appendiceal stump closure: a prospective randomized study. Surg Laparosc Endosc Percutan Tech.

[11] Rickert A (2012). Appendix stump closure with titanium clips in laparoscopic appendectomy. Langenbecks Arch Surg.

[12] Bratzler DW (2013). Clinical practice guidelines for antimicrobial prophylaxis in surgery. Surg Infect.

[13] Costanavarro D, Jiménezfuertes M, Illánriquelme A (2013). Laparoscopic appendectomy: quality care and cost-effectiveness for today’s economy. World J Emerg Surg: WJES.

[14] Gonenc M (2012). Intracorporeal knotting versus metal endoclip application for the closure of the appendiceal stump during laparoscopic appendectomy in uncomplicated appendicitis. J Laparoendosc Adv Surg Tech A.

[15] Gomes CA (2012). The appendiceal stump closure during laparoscopy: historical, surgical, and future perspectives. Surg Laparosc Endosc Percutan Tech.

[16] Allemann P (2011). Prevention of infectious complications after laparoscopic appendectomy for complicated acute appendicitis–the role of routine abdominal drainage. Langenbecks Arch Surg.

[17] Jani PG, Nyaga PN (2011). Peritoneal drains in perforated appendicitis without peritonitis: a prospective randomized controlled study. East Central Afr J Surg.

[18] Hanssen A, Plotnikov S, Dubois R (2007). Laparoscopic appendectomy using a polymetric clip to close the appendicular stump. JSLS.

[19] Tan TM, Okada M (1999). The efficiency of absorbable clips in minimally invasive surgery. Surg Today.

[20] Deans GT, Wilson MS, Brough WA (1995). The ability of laparoscopic clips to withstand high intraluminal pressure. Arch Surg.

[21] Klein RD (1994). Comparison of titanium and absorbable polymeric surgical clips for use in laparoscopic cholecystectomy. Surg Endosc.

[22] Alis H (2012). Metal endoclips for the closure of the appendiceal stump in laparoscopic appendectomy. Tech Coloproctol.

[23] Gomes CA (2013). Appendiceal stump closure by metal endoclip in the management of complicated acute appendicitis. World J Emerg Surg.

[24] Di SS (2014). A cost-effective technique for laparoscopic appendectomy: outcomes and costs of a case-control prospective single-operator study of 112 unselected consecutive cases of complicated acute appendicitis. J Am Coll Surg.

[25] Rembiasz K (2010). Analysis of complications of laparoscopic management of abdominal diseases related to extended indications. Videosurg Other Miniinvasive Tech.

